# A review of the clinical spectrum of *BRAT1* disorders and case of developmental and epileptic encephalopathy surviving into adulthood

**DOI:** 10.1016/j.ebr.2022.100549

**Published:** 2022-05-08

**Authors:** Ross Fowkes, Menatalla Elwan, Ela Akay, Clinton J Mitchell, Rhys H Thomas, David Lewis-Smith

**Affiliations:** a Department of Neurology, Royal Victoria Infirmary, Newcastle upon Tyne NE1 4LP, United Kingdom; b Department of Neurology, University Hospital of Wales, Heath Park, Cardiff CF14 4XN, United Kingdom; c Translational and Clinical Research Institute, Newcastle University, Newcastle upon Tyne NE2 4HH, United Kingdom; d Wellcome Centre for Mitochondrial Research, Newcastle University, Newcastle upon Tyne NE2 4HH, United Kingdom

**Keywords:** *BRAT1*, Epilepsy, Lethal neonatal rigidity and multifocal seizure syndrome, Epileptic encephalopathy, Adult

## Abstract

Pathogenic variants in *BRAT1* are associated with a spectrum of clinical syndromes ranging from Lethal Neonatal Rigidity and Multifocal Seizure syndrome (RMFSL) to Neurodevelopmental Disorder with Cerebellar Atrophy and with or without Seizures (NEDCAS). RMFSL is characterized by early-onset multifocal seizures with microcephaly. Death occurs during infancy although a less severe course with later onset seizures and longer survival into childhood has been described. Here, we summarize published cases of *BRAT1* disorders and present the case of a 20-year-old man with two heterozygous *BRAT1* variants and a relatively later age of seizure onset with survival into adulthood. This case expands the spectrum of disease associated with *BRAT1* variants and highlights the utility of genetic testing to identify the cause of developmental and epileptic encephalopathies where clinical heterogeneity within a spectrum of disease exists.

## Highlights

• We report a patient with a *BRAT1* disorder who survived into adulthood.
• Infantile-onset developmental and epileptic encephalopathy rather than an ataxic presentation was the presenting feature.
• Compound heterozygous *BRAT1* variants may be associated with later onset of seizures and longer survival reflecting a spectrum of clinical disease.

## 1 Introduction

Sporadic developmental and epileptic encephalopathies (DEE) are most commonly attributable to monoallelic variants arising *de novo* but can be transmitted with recessive and x-linked variants. Pathogenic biallelic variants in *BRAT1* are associated with a spectrum of disease including Lethal Neonatal Rigidity and Multifocal Seizure Syndrome (RMFSL, OMIM: 614498), Epilepsy of Infancy with Migrating Focal Seizures (EIMFS), and Neurodevelopmental Disorder with Cerebellar Atrophy and with or without Seizures (NEDCAS, OMIM: 618056) [1].

RMFSL is a severe disorder characterized by microcephaly, axial and appendicular rigidity, early-onset (possibly *in utero)* multifocal seizures, and episodes of bradycardia and apnea typically resulting in death during infancy [2], [3], [4]. A RMFSL-like phenotype has been described, with patients sharing some of the clinical features of RMFSL, but with a longer survival and later onset of epilepsy [5], [6], [7], [8]. Bialleic variants in *BRAT1* can also cause EIMFS, an early-onset encephalopathic syndrome characterized by seizures that appear to ‘migrate’ between cerebral hemispheres and significant cognitive impairment and developmental delay [9], [10]. Individuals with EIMFS caused by *BRAT1* variants may have an earlier onset and greater severity than those with EIMFS due to other causes [10]. RMFSL and EIMFS have genetic and phenotypic overlap and may represent similar disorders or different perspectives of the same disorder [11]. In contrast to these two, NEDCAS is a milder phenotype where patients may survive past infancy and exhibit intellectual disability, and ataxia with cerebellar degeneration with or without seizures [8], [12]. The current understanding of the spectrum of BRAT1 disorders reported in the literature is summarized in Table 1 which demonstrates the clinical, radiological, and electrophysiological heterogeneity seen in disorders caused by *BRAT1* variants.

**Table 1:** Previously reported cases of *BRAT1-*associated disorders.

	Case ID	*BRAT1* Variant	Age at onset of seizures	Seizure type	Syndrome	MRI head findings	EEG findings	Survival
Puffenberger, 2012 [2]	1	Homozygousc.638_639dupA, p. (Val214Glyfs*189)	Shortly after birth	Facial and arm jerks.	RMFSL	Normal or mild frontal hypoplasia	Bilateral medium–high voltage spikes over temporal and central regions, frequent multifocal seizures, background slowing, and no posterior rhythm.	< 4 months
	2							
	3							
Saunders, 2012 [3]	1	Homozygousc.453_454insATCTTCTC, p. (Leu152Ilefs*70)	Day 1	NA	RMFSL	Normal	Focal epileptiform and sharp wave activity.	NK
Saitsu, 2014 [21]	1	Compound heterozygous c.176 T > C, p.(Leu59Pro); c.962_963delTC, p.(Leu321Profs*81)	Day 7	Generalized tonic-clonic, Myoclonic	RMFSL	Cerebellar atrophy	Suppression-burst pattern.	21 months
	2		Day 1	Myoclonic, clonic and tonic.	RMFSL		Suppression-burst pattern.	3 months
Straussberg, 2015 [4]	1	Homozygousc.1177delG, p. (Ala393Leufs*3)	Day 1	Myoclonic	RMFSL	Normal	Sharp waves and bilateral spikes predominantly over the right hemisphere.	5 months
	2		Day 1	Myoclonic	RMFSL	Normal	Bilateral epileptic activity with bilateral discharges.	6 months
Van de Pol, 2015 [22]	1	NA	1st month	Tonic-clonic	RMFSL	Generalized atrophy	Severely abnormal background, multifocal sharp waves, and frequent multifocal epileptic seizure activity.	3 months
	2	Homozygousc.638dupA, p. (Val214Glyfs*189)	1st month	Myoclonic	RMFSL	Generalized atrophy	Continuous abnormal background pattern, multifocal seizure activity.	17 months
	3		1st month	Tonic, myoclonic	RMFSL	NA	Burst-suppression pattern with long suppressions, multifocal negative sharp waves.	2 months
Hanes, 2015 [5]	1	Compound heterozygousc.294dupA, p.(Leu99Thrfs*92) ; c.1825C > T, p.(Arg609Trp)	5 months	Focal with impaired awareness	NEDCAS	Cerebellar atrophy	Slow awake background. No epileptiform discharges.	Alive at 4 years
Fernández-Jáen, 2016 [23]	1	Compound heterozygous c.1564G > A, p.(Glu522Lys); c.638dupA, p.(Val214Glyfs*189)	None	None	NEDCAS	Cerebellar atrophy	Normal	Alive at 4.5 years
Horn, 2016 [6]	1	Compound heterozygousc.638dupA, p.(Val214Glyfs*189) ; c.1134 + 1G > A	5 months	Myoclonic	RMFSL	Thin corpus callosum, dilated lateral ventricles, delayed myelination.	Focal continuous spike discharges in the right more than in the left occipital region	5 years and 9 months
	2		Day 1	Myoclonic	RMFSL	NA	Multifocal sharp waves, focal status epilepticus in the right temporal posterior region	2 months
Mundy, 2016 [7]	1	Compound heterozygous c.294dupA, p.(Leu99Thrfs*92); c.1925C > A, p.(Ala642Glu)	3 months	NA	RMFSL	Thin corpus callosum, cerebellar hypoplasia.	Left- sided temporo-occipital epileptiform discharges and absence of a posterior dominant rhythm.	Alive at 6 years
Smith, 2016 [17]	1	Compound heterozygous c.1857G > A, p.(Trp619*); c.2125_2128delTTTG, p.(Phe709Thrfs*17)	4 months	Myoclonic, focal with impaired awareness, focal to bilateral tonic-clonic.	RMFSL	Global cerebral and cerebellar atrophy	Bilateral multifocal epileptiform activity	15 months
	2		9 months	Focal with impaired awareness, focal to bilateral tonic-clonic.	RMFSL	Normal	Bilateral multifocal epileptiform activity	Alive at 4 years and 4 months
Srivastava, 2016 [8]	1	Compound heterozygousc.638dupA, p.(Val214Glyfs*289) ; c.803 + 1G > C	No seizures reported	None	NEDCAS	Cerebellar atrophy	NA	Alive 10 years
	2		No seizures reported	None	NEDCAS	Cerebellar atrophy	Normal	Alive at 6 years
	3		3 years	Staring episodes	RMFSL	Cerebellar atrophy	Frequent 3–4 Hz generalized spike and wave complexes	Alive at 4 years and 4 months
	4		4 months	Tonic, focal status epilepticus	RMFSL	Mild cerebral atrophy	Generalized and focal bi-posterior quadrant slowing, and multi-focal epileptiform activity.	Alive at 15 months
Celik, 2017 [24]	1	Homozygousc.2230_2237dupAACACTGC, p. (Ser747Thrfs*36)	1st month	Myoclonic	RMFSL	Cerebellar atrophy, thinning of corpus callosum	Background activity of – 6 Hz theta, bilateral frontotemporal sharp waves, and 8–10 Hz ictal rhythm during clinical seizures.	10 months
Hegde, 2017 [25]	1	Homozygous c.617 T > A, p.(Leu206*)	Day 3	Clonic	RMFSL	Cortical and cerebellar atrophy	Occasional generalized bursts of epileptiform activity with relatively well-preserved background activity, burst suppression pattern.	4 months
Skafi, 2018 [26]	1	Homozygous c.638_639dupA, p.(Val214Glyfs*189)	Day 1	Myoclonic	RMFSL	Normal	Low-voltage background without epileptic discharges.	3 months
Szymańska, 2018 [16]	1	Homozygousc.1313_1314delAG, p. (Gln438Argfs*51)	Day 1	Myoclonic	RMFSL	Cerebral atrophy, thinning of corpus callosum	NA	6 months
	2		Day 1	Myoclonic, tonic, clonic	RMFSL	Widened subarachnoid space	Generalized and focal sharp and spike waves.	12 months
Van Ommeren, 2018 [27]	1	Homozygous c.1395G > C, p.(Thr465Thr)	Day 1	Myoclonic	RMFSL	Thinning of corpus callosum	Diffuse encephalopathy, with frequent ictal activity from multiple cortical areas.	2 months
Mahjoub, 2019 [28]	1	Homozygous c.185 T > A, p.(Val62Glu)	No seizures reported	None	NEDCAS	Cerebellar atrophy	NA	Alive at 24 years
	2		No seizures reported	None	NEDCAS	Cerebellar atrophy	NA	Alive at 7 years
Colak, 2020 [29]	2	Homozygous c.1395G > C, p.(Thr465Thr)	No seizures reported	None	NEDCAS	Cerebellar atrophy	Generalized epileptiform activity, migrating focal epileptiform activity, background deceleration.	Alive at 7 years
Pourahmadiyan, 2020 [30]	1	Homozygous c.2041G > T, p.(E681*)	NK	Focal seizures	RMFSL	NA	NA	6 days
Scheffer, 2020 [10]	1	Compound heterozygous c.964C > T, p.(Gln322*); c.2284C > T, p.(Gln762*)	Day 1	Focal motor migrating between hemispheres	EIMFS	Underopercularization of the Sylvian fissure	Multifocal epileptiform discharges, discontinuous background Ictal: focal seizure migrating from left central region to right hemisphere.	1 month
	2		Day 1	Focal clonic seizures with apnea and tachycardia migrating between hemispheres	EIMFS	Thinning of corpus callosum	Multifocal epileptiform discharges, discontinuous background intermittently Ictal: migrating focal seizures; central, right occipital spread to left occipital region, left temporal spread to left hemisphere then right hemisphere, bi-occipital onset.	10 months
	3	Homozygous c.1498 + 1G > A	Day 1	Focal, multifocal motor seizures with clonic features, apnea, eye deviation epileptic spasms; tonic seizures	EIMFS	White matter volume loss	Multifocal epileptiform discharges Ictal: migrating focal seizures; seizures arising from right central region, vertex, left central, left occipital, right temporal, and left temporal region. Epileptic spasms and periodic spasms, hypsarrhythmia	4 years and 3 months
	4	Homozygous c.1120G > T, p.(Glu374*)	Day 4	Focal motor clonic seizures, migrating between hemispheres	EIMFS	Subdural hemorrhage	Multifocal epileptiform discharges, discontinuous background Ictal: myoclonic seizures, clonic seizures, facial clonic movements, with migration from right posterior occipital region to left posterior region.	2 months
	5	Compound Heterozygousc.1359_1361delCCT, p.(Leu454del) ; c.1395G > C, p.(Thr465Thr)	Day 1	Focal seizures migrating between hemispheres	EIMFS	Subdural hemorrhage	Multifocal epileptiform discharges, discontinuous background Ictal: migrating focal seizures from one region to another, most frequent onset from right posterior quadrant, other onsets in left posterior region and left fronto-central region.	14 months
Balasundaram, 2021 [31]	1	Bilalelic deletion of at least exons 1–2	Day 1	Tonic-clonic, myoclonic	RMFSL	Corpus callosum thinning	Bilateral medium–high voltage spikes over temporal and central regions, frequent multifocal seizures, background slowing, and no posterior rhythm.	2.5 months
Li, 2021 [32]	1	Homozygous c.233G > C, p.(Arg78Pro)	1 month	Myoclonic	RMFSL	Corpus callosum thinning	Sharp wave discharges in the left forehead-parietal region than in the right forehead- parietal region.	7 months
Stödberg, 2020 [18]	1	Compound heterozygous c.1771‐1G > C; p.? c.294dup; p.(Leu99Thrfs*92)	1 month	NA	NA	Pachygyria	NA	NA
	2		1 month	NA	NA	Pachygyria	NA	NA
	3		1 month	NA	NA	Pachygyria	NA	NA
Nuovo, 2022 [33]	1	Compound heterozygousc.638dup, p.(Val214GlyfsTer189) ; c.1395G > A, p.(Thr465 = )	No seizures reported	NA	NEDCAS	Mildly shrunken cerebellum with enlarged interfolial spaces	NA	Alive at 15 years
	2		No seizures reported	NA	NEDCAS	Widened cerebellar interfolial spaces	NA	Alive at 10 years
	3		14 years	Tonic-clonic, staring spells, paroxysmal blinking	NEDCAS	Shrunken cerebellum with reduction on N-acetyl aspartate peak	Diffuse sharp and wave complexes	Alive at 18 years
Present case	1	Suspected compound heterozygous c.294dupA, p.(Leu99Thrfs*92); c.1925C > A, p.(Ala642Glu)	8 months	Tonic-clonic, myoclonic, suspected absence	RMFSL	Cerebellar hypoplasia	Symmetric background alpha, beta, and theta rhythms with occasional increased amplitude delta activity, which was notched and sharp. There were also frequent focal sharp wave and spike discharges, both simultaneously and independently over both hemispheres	20 years

NA – not available. Median age at seizure onset: 1st day of life, Range: 0–1094, Interquartile range: 1–30.

We describe the history of a man with *BRAT1* variants and an RMFSL-like phenotype with microcephaly, limb hypertonia, developmental delay, and multifocal epilepsy. Significantly, he survived until the age of twenty years, substantially longer than any previously reported case.

## 2 Case report

A 20-year-old man with non-consanguineous parents was born by caesarean section at 35 weeks due to severe intrauterine growth restriction. His mother and father did not have any history of epilepsy, neurological disorders, or any other medical problems.

His birth weight was 1215 g and his head circumference was 27.2 cm, both below the third centile. He spent four weeks on the neonatal unit due to low birth weight and was noted to have abdominal distention, axial hypotonia, limb hypertonia and episodes of both bradycardia and apnea. At the age of one year, he still exhibited axial hypotonia and limb hypertonia in addition to horizontal and upbeat nystagmus, a sluggish pupillary reflex to light, and inability fix his gaze on objects. He had persistent constipation requiring treatment with regular laxatives and enemas. A viral serology screen did not demonstrate evidence of congenital viral infection with CMV, parvovirus B19, Rubella, HSV, or congenital toxoplasmosis.

He had severe global developmental delay without regression. He did not smile until the age of 16 months, made babbling noises at the age of 21 months, and rolled at 22 months. He was never able to walk and required a wheelchair to mobilize. He had a wide nasal bridge, bilateral epicanthic folds, and dysmorphic ears.

At 8 months of age, he presented with an afebrile transient loss of consciousness lasting 5 min. He was reported to have been unresponsive, grinding his teeth with his eyes rolled backwards, and was grey and floppy without limb movements.

By the age of 3 years, his parents had witnessed at least six unresponsive, cyanotic, and hypotonic episodes with twitching of his limbs lasting for a few minutes each which were presumed to be bilateral tonic-clonic seizures of unknown onset. This was in addition to multiple daily brief episodes of apparent loss of awareness and occasional myoclonic jerks. At this point, epilepsy was diagnosed, and he commenced sodium valproate 200 mg twice a day. After two-years without tonic-clonic seizures, these relapsed, increasing in frequency to clusters of 3–4 seizures occurring approximately once a week. We have not been able to obtain medical records or a collateral history covering the period from childhood to until he represented at the age of 15 years, when he commenced topiramate but continued to have seizures weekly. This was thought to be contributed to, in part, by non-concordance with anti-seizure medication when he stopped eating and drinking. A gastrostomy tube was subsequently inserted to support nutrition and medication administration. Despite this, seizure frequency worsened over the following five years, and at the age of 20, levetiracetam was added with an improvement in seizure frequency. He died aged 20 years due to aspiration pneumonia complicating unexplained gastroparesis with vomiting.

Blood tests in early childhood including full blood count, urea and electrolytes, and liver and thyroid profiles were normal, as were serum lactate and pyruvate. While the original data from electroencephalography (EEG) study at four years of age is no longer available, it was reported as showing symmetric background alpha, beta, and theta rhythms with occasional increased amplitude delta activity, which was notched and sharp. There were also frequent focal sharp wave and spike discharges, both simultaneously and independently over both hemispheres, consistent with multifocal epilepsy. The report does not mention capture of the events characterized by loss of awareness, that we classify as unknown onset behavior arrest seizures on purely clinical grounds. Similarly, the original magnetic resonance images acquired the same year are no longer available for review but were reported as showing cerebellar hypoplasia with a prominent vermis. EEG during his terminal illness in the intensive care unit without sedation was consistent with severe encephalopathy, demonstrating widespread low amplitude theta and delta rhythms without reactivity to auditory stimuli.

Genetic testing yielded a normal karyotype with no evidence of fragile X syndrome. Whole exome sequencing was preformed during his terminal admission at 20 years of age, and reported as an *in silico* panel of selected genes (gene list provided as a supplement) by GEMINI (Cambridge University Hospitals NHS Foundation Trust). This revealed two variants in *BRAT1*, c.294dupA, p.(Leu99Thrfs*92) and c.1925C>A, p.(Ala642Glu). Both variants have been interpreted as pathogenic in previous publications and are present in gnomAD (p.Leu99Thrfs*92 on 42 occasions and p.Ala642Glu 8 times) below the maximum frequency that would refute their pathogenicity in a recessive disorder and neither has been reported in the homozygous state in gnomAD [7], [13], [14]. We interpret them according to Amercan College of Medical Genetics and Genomics criteria as pathogenic and likely pathogenic respectively [15]. The lack of parental DNA and the distance between variants has meant that it has not been possible to confirm that these variants are in *trans* (compound heterozygous).

## 3 Discussion

This case illustrates some important clinical lessons applicable to the genetic investigation of epilepsy in adults. First, recessive forms of DEE may be identified with panel testing in adults, even without consanguinity. However, compared to pediatric patients, adults may be less likely to have parental DNA available to confirm the suspected genetic mechanism through segregation of variants; this is also a challenge to the interpretation of monoallelic potentially *de novo* variants. In the very near future, advances in genetic sequencing technologies will help with the interpretation of variants in recessive disease genes where segregation cannot be demonstrated. In this case, the variants were 5 kilobases apart, raising the possibility that a single long-read may span both loci. Second, among the increasing number of young adults transitioning to adult services with DEE, some have disorders that were previously not recognized in adulthood due to the biases of genetic discovery and initial phenotypic studies, which focus on pediatric cohorts, often comprising children with particularly severe phenotypes. Consequently, some genetic causes may thus far have been associated only with the more severe extreme of their latent clinical spectrum. Finally, for adult patients, the threshold for testing should take account of the clinical heterogeneity of each gene, and the possible incompleteness of historical records of clinical phenomenology, development, EEG, and MRI. These may have been destroyed or incomplete, or not followed the patient from hospital to hospital. When assessing an adult with early onset epilepsy, the historical records and previous investigations are likely to predate the discovery of the particular cause of their epilepsy. Accordingly, the absence of the corresponding classic gene-phenotype association in these medical records should not be assumed to mean that the phenotype was never present — it may not have been actively sought.

The *BRAT1* gene (7p22.3) encodes the BRCA1-associated ATM activator 1 protein. This protein is involved in the response to DNA damage and apoptosis and in the regulation of mitochondrial homeostasis [10]. Few detailed descriptions of people with disorders attributable to *BRAT1* variants have been published, but these have revealed heterogeneous phenotypic constellations with a varying degree of clinical severity. If seizures are present, they are typically but not exclusively seen in early infancy. The median age of seizure onset in the reported cases of *BRAT1*-related disease is the first day of life (Table 1).

At the most severe end of the clinical spectrum of disease is RMFSL, typified by severe drug-resistant seizures beginning *in utero* or within the first days of life, microcephaly, rigidity, swallowing difficulties, and poor psychomotor development. Infants with RMFSL experience apneic and bradycardic episodes culminating in cardiorespiratory arrest and death, typically within the first year of life [5].

A less severe clinical course is characterized by features similar to RMFSL but with survival past infancy, later onset epilepsy and continued development [5], [6], [7], [8]. Our case matches this phenotype, with evidence of microcephaly, limb hypertonia, severe global developmental impairment and drug resistant multifocal epilepsy, and represents the longest reported survival in such an individual with this phenotype to date: the previous oldest patient with this phenotype was reported to still be alive at the age of six years and could be as old as 11 now [7]. The mildest reported constellation of *BRAT1*-related disease is characterized by intellectual disability, ataxia, and cerebellar atrophy with or without microcephaly and epilepsy [8], [12].

It appears that the severity of disease is linked to zygosity of the *BRAT1* variants with most of the reported homozygous cases developing seizures immediately after birth or suspected to have had seizures *in utero*. The longest reported survival for a homozygous patient is 12 months [16]. The reported cases with compound heterozygous *BRAT1* variants, however, show a relatively delayed onset of seizures, typically within the first few months of life but in a few cases, after one year of age. These patients generally survive longer, with several previously reported cases of survival into childhood and the present case showing that survival into adulthood is possible. However, two sets of siblings with compound heterozygous variants have shown significant phenotypic variability, indicating that genetic expressivity is variable and other genetic or non-genetic factors contribute [6], [17]. Overall, it appears that *BRAT1-*related epilepsies can include both focal-onset and generalized-onset seizures. Beyond the individual we present here with unknown onset for seizures associated with behavioral arrest, only one other has been reported to have seizures compatible with absences (case 3 in Srivastava, 2016 [7]). This person had late onset epilepsy given the of context of *BRAT1-*related disorder (3 years) and survival to at least 4 years. This may reflect the difficulty of detecting non-motor seizures in infancy or the increased propensity for absences to occur as the brain develops towards mid-childhood (onset of absences before 4 years of age is rare across the epilepsies and should prompt consideration of genetic testing). In either case, the recognition of non-motor seizures in a person with *BRAT1*-related epilepsy may be a marker of survival beyond infancy, and thus a milder form of these diseases.

A person with identical *BRAT1* variants has previously been described by Mundy *et al.,* who were able to confirm compound heterozygosity and emphasized survival beyond infancy [7]. Similarly, to the present case, they describe microcephaly, mild dysmorphic features, abnormal tone, and developmental delay in addition to drug-resistant seizures. However, the individual that they reported had developed seizures at a younger age (3 months vs 8 months). The three *BRAT1* cases reported in Stödberg *et al* all had shared the c.294dupA, p.(Leu99Thrfs*92) genotype with our case; all three were reported to have pachygyria – not reported in the individual we report, for whom we were not able to review the original MRI or repeat the study [18]. The other variant we report here, c.1925C>A, p.(Ala642Glu), has been described in compound heterozygosity in someone with a neurodevelopmental syndrome with progressive neurodegeneration that fits neither the syndromes of EIMFS nor RMFLS, and who had focal impaired awareness seizures [7]. It is possible that the events with apparent loss of awareness of the person we report were focal impaired awareness seizures.

The prevalence of epilepsy due to *BRAT1* variants in adults is currently unknown, although none was detected among 150 adult patients with neurodevelopmental disorders with epilepsy (using a gene panel that included *BRAT1*) [19]. The national screening study of children with seizures in Scotland did not include *BRAT1* on its panel [20].

## 4 Conclusion

Biallelic pathogenic *BRAT1* variants cause a spectrum of clinical disease with and without seizures. With wider testing and a higher index of suspicion, we may see cases with longer survival in the context of a later age of seizure onset and compound heterozygosity. This case expands the clinical spectrum further to include people reaching adulthood with multifocal epilepsy, dysmorphic features, and developmental delay.

## Declaration of Competing Interest

The authors declare the following financial interests/personal relationships which may be considered as potential competing interests: R.H.T. reports Honoraria from Arvelle, Bial, Eisai, GW Pharma, Sanofi, UCB Pharma, UNEEG and Zogenix. E.A. reports Honoraria from Arvelle. R.F., M.E., C.J.M., and D.L.-S. have nothing to declare.

## References

[1] International League Against Epilepsy. Epilepsy of Infancy with Migrating Focal Seizures n.d. https://www.epilepsydiagnosis.org/syndrome/infancy-migrating-focal-overview.html (accessed February 17, 2022).

[2] Puffenberger EG, Jinks RN, Sougnez C, Cibulskis K, Willert RA, Achilly NP, et al. Genetic Mapping and Exome Sequencing Identify Variants Associated with Five Novel Diseases. PLoS ONE. 2012. 7. e28936. https://doi.org/10.1371/journal.pone.0028936. 10.1371/journal.pone.0028936 PMC3260153 22279524

[3] Saunders C.J., Miller N.A., Soden S.E., Dinwiddie D.L., Noll A., Alnadi N.A. (2012). Rapid whole-genome sequencing for genetic disease diagnosis in neonatal intensive care units. Sci Transl Med.

[4] Straussberg R., Ganelin-Cohen E., Goldberg-Stern H., Tzur S., Behar D.M., Smirin-Yosef P. (2015). Lethal neonatal rigidity and multifocal seizure syndrome–report of another family with a BRAT1 mutation. Eur J Paediatr Neurol EJPN Off J Eur Paediatr Neurol Soc.

[5] Hanes I., Kozenko M., Callen D.J.A. (2015). Lethal Neonatal Rigidity and Multifocal Seizure Syndrome—A Misnamed Disorder?. Pediatr Neurol.

[6] Horn D., Weschke B., Knierim E., Fischer-Zirnsak B., Stenzel W., Schuelke M. (2016). BRAT1 mutations are associated with infantile epileptic encephalopathy, mitochondrial dysfunction, and survival into childhood. Am J Med Genet A.

[7] Mundy S.A., Krock B.L., Mao R., Shen J.J. (2016). BRAT1-related disease-identification of a patient without early lethality. Am J Med Genet A.

[8] Srivastava S., Olson H.E., Cohen J.S., Gubbels C.S., Lincoln S., Davis B.T. (2016). BRAT1 mutations present with a spectrum of clinical severity. Am J Med Genet A.

[9] Burgess R., Wang S., McTague A., Boysen K.E., Yang X., Zeng Q.i. (2019). The Genetic Landscape of Epilepsy of Infancy with Migrating Focal Seizures. Ann Neurol.

[10] Scheffer I.E., Boysen K.E., Schneider A.L., Myers C.T., Mehaffey M.G., Rochtus A.M. (2020). BRAT1 encephalopathy: a recessive cause of epilepsy of infancy with migrating focal seizures. Dev Med Child Neurol.

[11] Guerrini R. Epilepsy of infancy with migrating focal seizures or rigidity and multifocal seizure syndrome, lethal neonatal? Different emphases on a severe phenotype. Dev Med Child Neurol. 2020. 62. 1012–1012. https://doi.org/10.1111/dmcn.14445. 10.1111/dmcn.14445 31879938

[12] Valence S., Cochet E., Rougeot C., Garel C., Chantot-Bastaraud S., Lainey E. (2019). Exome sequencing in congenital ataxia identifies two new candidate genes and highlights a pathophysiological link between some congenital ataxias and early infantile epileptic encephalopathies. Genet Med.

[13] Whiffin N., Minikel E., Walsh R., O’Donnell-Luria A.H., Karczewski K., Ing A.Y. (2017). Using high-resolution variant frequencies to empower clinical genome interpretation. Genet Med.

[14] About gnomAD | gnomAD n.d. https://gnomad.broadinstitute.org/about. (accessed September 15, 2021).

[15] Richards S., Aziz N., Bale S., Bick D., Das S., Gastier-Foster J. (2015). Standards and guidelines for the interpretation of sequence variants: a joint consensus recommendation of the American College of Medical Genetics and Genomics and the Association for Molecular Pathology. Genet Med Off J Am Coll Med Genet.

[16] Szymańska K., Laure-Kamionowska M., Szczałuba K., Koppolu A., Furmanek M., Kuśmierska K. (2018). Clinico-pathological correlation in case of BRAT1 mutation. Folia Neuropathol.

[17] Smith N.J., Lipsett J., Dibbens L.M., Heron S.E. (2016). BRAT1 -associated neurodegeneration: Intra-familial phenotypic differences in siblings. Am J Med Genet A.

[18] Stödberg T., Tomson T., Barbaro M., Stranneheim H., Anderlid B.-M., Carlsson S. (2020). Epilepsy syndromes, etiologies, and the use of next-generation sequencing in epilepsy presenting in the first 2 years of life: A population-based study. Epilepsia.

[19] Zacher P., Mayer T., Brandhoff F., Bartolomaeus T., Le Duc D., Finzel M. (2021). The genetic landscape of intellectual disability and epilepsy in adults and the elderly: a systematic genetic work-up of 150 individuals. Genet Med.

[20] Symonds JD, Zuberi SM, Stewart K, McLellan A, O’Regan M, MacLeod S, et al. Incidence and phenotypes of childhood-onset genetic epilepsies: a prospective population-based national cohort. Brain J Neurol. 2019. 142. 2303–18. https://doi.org/10.1093/brain/awz195. 10.1093/brain/awz195 PMC6658850 31302675

[21] Saitsu H., Yamashita S., Tanaka Y., Tsurusaki Y., Nakashima M., Miyake N. (2014). Compound heterozygous BRAT1 mutations cause familial Ohtahara syndrome with hypertonia and microcephaly. J Hum Genet.

[22] van de Pol L., Wolf N., van Weissenbruch M., Stam C., Weiss J., Waisfisz Q. (2015). Early-Onset Severe Encephalopathy with Epilepsy: The BRAT1 Gene Should Be Added to the List of Causes. Neuropediatrics.

[23] Fernández-Jaén A., Álvarez S., Young So E., Ouchi T., Jiménez de la Peña M., Duat A. (2016). Mutations in BRAT1 cause autosomal recessive progressive encephalopathy: Report of a Spanish patient. Eur J Paediatr Neurol EJPN Off J Eur Paediatr Neurol Soc.

[24] Celik Y., Okuyaz C., Arslankoylu A.E., Ceylaner S. (2017). Lethal neonatal rigidity and multifocal seizure syndrome with a new mutation in BRAT1. Epilepsy Behav Case Rep.

[25] Hegde A., Sanghvi K., Kamavat P., Jalan A. (2017). BRCA1-associated ataxia telangiectasia mutated activation-1 mutation: An addition to the early infantile epileptic encephalopathy panel. J Clin Neonatol.

[26] O S., A F., Ba M., H J., S M., R H. (2018). Rigidity with Multifocal Seizure Syndrome, Lethal Neonatal in a Lebanese Neonate. A Rare Case Report J Pediatr Disord Neonatal Care.

[27] Van Ommeren R.H., Gao A.F., Blaser S.I., Chitayat D.A., Hazrati L.-N. (2018). BRAT1 Mutation: The First Reported Case of Chinese Origin and Review of the Literature. J Neuropathol Exp Neurol.

[28] Mahjoub A, Cihlarova Z, Tétreault M, MacNeil L, Sondheimer N, Caldecott KW, et al. Homozygous pathogenic variant in BRAT1 associated with nonprogressive cerebellar ataxia. Neurol Genet. 2019. 5. e359. https://doi.org/10.1212/NXG.0000000000000359. 10.1212/NXG.0000000000000359 PMC6773431 31742228

[29] Colak F.K., Guleray N., Azapagasi E., Yazıcı M.U., Aksoy E., Ceylan N. (2020). An intronic variant in BRAT1 creates a cryptic splice site, causing epileptic encephalopathy without prominent rigidity. Acta Neurol Belg.

[30] Pourahmadiyan A., Heidari M., Shojaaldini Ardakani H., Noorian S., Savad S. (2021). A novel pathogenic variant of *BRAT1* gene causes rigidity and multifocal seizure syndrome, lethal neonatal. Int J Neurosci.

[31] Balasundaram P., Fijas M., Nafday S. (2021). A Rare Case of Lethal Neonatal Rigidity and Multi-Focal Seizure Syndrome. Cureus.

[32] Li W., Wu S., Xu H., Zhao X., Pan Y., Huang H. (2022). Novel variant in BRAT1 with the lethal neonatal rigidity and multifocal seizure syndrome. Pediatr Res.

[33] Nuovo S., Baglioni V., De Mori R., Tardivo S., Caputi C., Ginevrino M. (2022). Clinical variability at the mild end of BRAT1-related spectrum: Evidence from two families with genotype-phenotype discordance. Hum Mutat.

