# Supplementary material for: A review of the clinical spectrum of BRAT1 disorders and case of developmental and epileptic encephalopathy surviving into adulthood
Source: Epilepsy Behav Rep. 2022 May 8;19:100549. doi: 10.1016/j.ebr.2022.100549 (PMC9126772; doi:10.1016/j.ebr.2022.100549)
Supplement: Supplementary data 1 [file mmc1.docx]

Gemini Epileptic Encephalopathy Gene Panel

ACY1

ADSL

ALDH7A1

ALG13

AMT

ANKRD11

ARHGEF9

ARID1A

ARID1B

ARX

ASXL3

ATP1A2

ATP1A3

ATP6AP2

ATRX

BRAT1

CACNA1A

CACNA1E

CACNA1H

CACNB4

CASK

CDKL5

CHD2

CHRNA2

CHRNA4

CHRNA7

CHRNB2

CLCN2

CLN3

CLN5

CLN6

CLN8

CNTNAP2

COL4A3BP

CPT2

CTSD

DCX

DDX3X

DEPDC5

DHDDS

DNM1

DOCK7

DYNC1H1

DYRK1A

EEF1A2

EFHC1

EHMT1

ELP4

FOLR1

FOXG1

GABBR2

GABRA1

GABRA1

GABRB3

GABRG2

GAMT

GCSH

GFAP

GLDC

GLUD1

GNAO1

GPHN

GRIN1

GRIN2A

GRIN2B

GRIN2B

HADH

HCN1

HNRNPU

HUWE1

IQSEC2

ITPA

KANSL1

KCNA1

KCNA2

KCNB1

KCNH1

KCNJ10

KCNMA1

KCNQ2

KCNQ3

KCNT1

KCTD7

KIAA1279

LGI1

LIAS

MAGI2

MAPK10

MBD5

MECP2

MEF2C

MFSD8

MTHFR

MTOR

NEXMIF

NPR2

NRXN1

NTRK2

PCDH19

PHGDH

PIGA

PLCB1

PNKP

PNPO

POLG

PPT1

PRRT2

PURA

QARS

RELN

RNASEH2A

RNASEH2B

RNASEH2C

RYR3

SAMHD1

SCN1A

SCN1B

SCN2A

SCN8A

SCN9A

SGCE

SLC12A5

SLC13A5

SLC16A2

SLC19A3

SLC1A3

SLC25A22

SLC2A1

SLC35A2

SLC6A1

SLC6A8

SLC9A6

SMARCA2

SMARCA4

SMARCB1

SMARCE1

SMC1A

SNAP25

SPTAN1

ST3GAL3

STX1B

STXBP1

SYN1

SYNGAP1

SYNJ1

TBC1D24

TBCE

TCF4

TPP1

TREX1

TSC1

TSC2

UBE2A

UBE3A

WDR45

WWOX

ZEB2
